# Author Correction: Evidence for multi-fragmentation and mass shedding of boulders on rubble-pile binary asteroid system (65803) Didymos

**DOI:** 10.1038/s41467-024-54185-2

**Published:** 2024-11-19

**Authors:** M. Pajola, F. Tusberti, A. Lucchetti, O. Barnouin, S. Cambioni, C. M. Ernst, E. Dotto, R. T. Daly, G. Poggiali, M. Hirabayashi, R. Nakano, E. Mazzotta Epifani, N. L. Chabot, V. Della Corte, A. Rivkin, H. Agrusa, Y. Zhang, L. Penasa, R.-L. Ballouz, S. Ivanovski, N. Murdoch, A. Rossi, C. Robin, S. Ieva, J. B. Vincent, F. Ferrari, S. D. Raducan, A. Campo-Bagatin, L. Parro, P. Benavidez, G. Tancredi, Ö. Karatekin, J. M. Trigo-Rodriguez, J. Sunshine, T. Farnham, E. Asphaug, J. D. P. Deshapriya, P. H. A. Hasselmann, J. Beccarelli, S. R. Schwartz, P. Abell, P. Michel, A. Cheng, J. R. Brucato, A. Zinzi, M. Amoroso, S. Pirrotta, G. Impresario, I. Bertini, A. Capannolo, S. Caporali, M. Ceresoli, G. Cremonese, M. Dall’Ora, I. Gai, L. Gomez Casajus, E. Gramigna, R. Lasagni Manghi, M. Lavagna, M. Lombardo, D. Modenini, P. Palumbo, D. Perna, P. Tortora, M. Zannoni, G. Zanotti

**Affiliations:** 1 INAF-Astronomical Observatory of Padova, Padova, Italy; 2 Johns Hopkins University Applied Physics Laboratory, Laurel, MD USA; 3 Department of Earth, Atmospheric and Planetary Sciences, Massachussets Institute of Technology, Cambridge, MA USA; 4 INAF-Osservatorio Astronomico di Roma, Roma, Italy; 5 INAF-Osservatorio Astrofisico di Arcetri, Firenze, Italy; 6 LESIA-Observatorie de Paris PSL, Paris, France; 7 Georgia Institute of Technology, Atlanta, GA USA; 8 Department of Aerospace Engineering, Auburn University, Auburn, AL USA; 9 INAF-Osservatorio Astronomico di Capodimonte, Napoli, Italy; 10 Department of Astronomy, University of Maryland, College Park, MD USA; 11 Université Côte d’Azur, Observatoire de la Côte d’Azur, CNRS, Laboratoire Lagrange, Nice, France; 12 Climate & Space Sciences and Engineering, University of Michigan, Hayward, MI USA; 13 INAF-Osservatorio Astronomico di Trieste, Trieste, Italy; 14 Institut Supérieur de l’Aéronautique et de l’Espace (ISAE-SUPAERO), Université de, Toulouse, France; 15 IFAC-CNR, Sesto Fiorentino, Firenze, Italy; 16 DLR Berlin, Berlin, Germany; 17 Dipartimento di Scienze e Tecnologie Aerospaziali, Politecnico di Milano—Bovisa Campus, Milano, Italy; 18 Space Research and Planetary Sciences, Physikalisches Institut, University of Bern, Bern, Switzerland; 19 Universidad de Alicante, de Alicante, Spain; 20 University of Arizona, Tucson, AZ USA; 21 Dpto. Astronomia, Facultad Ciencias Igua, Montevideo, Uruguay; 22 Royal Observatory of Belgium, Uccle, Belgium; 23 Institute of Space Sciences (ICE, CSIC) and Institut d’Estudis Espacials de Catalunya (IEEC), Barcelona, Spain; 24 Planetary Science Institute; University of Arizona, Tucson, AZ USA; 25 NASA Johnson Space Center, Houston, TX USA; 26 Department of Systems Innovation, School of Engineering, The University of Tokyo, Tokyo, Japan; 27 Agenzia Spaziale Italiana, Roma, Italy; 28 Space Science Data Center—ASI, Roma, Italy; 29 Dipartimento di Scienze & Tecnologie, Università degli Studi di Napoli “Parthenope”, Napoli, Italy; 30 Dipartimento di Ingegneria Industriale, Alma Mater Studiorum—Università di Bologna, Forlì, Italy; 31 Centro Interdipartimentale di Ricerca Industriale Aerospaziale, Alma Mater Studiorum—Università di Bologna, Forlì, Italy; 32 INAF-Istituto di Astrofisica e Planetologia Spaziali, Roma, Italy

**Keywords:** Asteroids, comets and Kuiper belt, Geomorphology, Structural geology

Correction to: *Nature Communications* 10.1038/s41467-024-50148-9, published online 30 July 2024

In this article the funding from the Spanish project PID2021-128062NB-I00 funded by MCIN/AEI was omitted. The original article has been corrected.

