# Supplementary material for: Evidence for multi-fragmentation and mass shedding of boulders on rubble-pile binary asteroid system (65803) Didymos
Source: Nat Commun. 2024 Jul 30;15:6205. doi: 10.1038/s41467-024-50148-9 (PMC11289111; doi:10.1038/s41467-024-50148-9)
Supplement: Supplementary file 1 — Supplementary Information [file 41467_2024_50148_MOESM1_ESM.pdf]

Supplementary Information

Evidence for multi-fragmentation and mass shedding of boulders on rubble-pile binary asteroid system (65803) Didymos

M. Pajola[1,*], F. Tusberti[1], A. Lucchetti[1], O. Barnouin[2], S. Cambioni[3], C.M. Ernst[2], E. Dotto[4], R.T. Daly[2], G. Poggiali[5,6], M. Hirabayashi[7], R. Nakano[7,8], E. Mazzotta Epifani[4], N.L. Chabot[2], V. Della Corte[9], A. Rivkin[2], H. Agrusa[10,11], Y. Zhang[12], L. Penasa[1], R.-L. Ballouz[2], S. Ivanovski[13], N. Murdoch[14], A. Rossi[15], C. Robin[14], S. Ieva[4], J.B. Vincent[16], F. Ferrari[17], S.D. Raducan[18], A. Campo-Bagatin[19], L. Parro[19,20], P. Benavidez[19], G. Tancredi[21], Ö. Karatekin[22], J.M. Trigo-Rodriguez[23], J. Sunshine[10], T. Farnham[10], E. Asphaug[24], J.D.P. Deshapriya[4], P.H.A. Hasselmann[4], J. Beccarelli[1], S.R. Schwartz[24], P. Abell[25], P. Michel[11,26], A. Cheng[2], J.R. Brucato[5], A. Zinzi[27,28], M. Amoroso[27], S. Pirrotta[27], G. Impresario[27], I. Bertini[29], A. Capannolo[14], S. Caporali[5], M. Ceresoli[17], G. Cremonese[1], M. Dall'Ora[9], I. Gai[30], L. Gomez Casajus[30], E. Gramigna[30], R. Lasagni Manghi[30], M. Lavagna[17], M. Lombardo[30], D. Modenini[30], P. Palumbo[31], D. Perna[4], P. Tortora[30], M. Zannoni[30], G. Zanotti[17].

[1]INAF-Astronomical Observatory of Padova, Vic. Osservatorio 5, 35122 Padova, Italy (*Corresponding author: maurizio.pajola@inaf.it);
[2]Johns Hopkins University Applied Physics Laboratory, Laurel, MD 20723, USA;
[3]Dept. of Earth, Atmospheric and Planetary Sciences, Massachussets Institute of Technology, Cambridge, MA-USA;
[4]INAF-Osservatorio Astronomico di Roma, Monte Porzio Catone, Roma, Italy;
[5]INAF-Osservatorio Astrofisico di Arcetri, Firenze, Italy;
[6]LESIA-Observatorie de Paris PSL, Paris, France;
[7]Georgia Institute of Technology, Atlanta, GA 30332, USA;
[8]Department of Aerospace Engineering, Auburn University, AL 36849, USA;
[9]INAF-Osservatorio Astronomico di Capodimonte, Napoli, Italy;
[10]University of Maryland, Department of Astronomy, MD-USA;
[11]Université Côte d'Azur, Observatoire de la Côte d'Azur, CNRS, Laboratoire Lagrange, Nice, France;
[12]Climate & Space Sciences and Engineering, University of Michigan, MI-USA;
[13]INAF-Osservatorio Astronomico di Trieste, Trieste, Italy;
[14]Institut Supérieur de l'Aéronautique et de l'Espace (ISAE-SUPAERO), Université de Toulouse, France;
[15]IFAC-CNR, Sesto Fiorentino, Firenze, Italy;
[16]DLR Berlin, Germany;
[17]Politecnico di Milano - Bovisa Campus, Dipartimento di Scienze e Tecnologie Aerospaziali, Milano, Italy;
[18]Space Research and Planetary Sciences, Physikalisches Institut, University of Bern, Bern, Switzerland;
[19]Universidad de Alicante, Spain;
[20]University of Arizona, AZ-USA;
[21]Dpto. Astronomia, Facultad Ciencias Igua 4225, Montevideo, Uruguay;
[22]Royal Observatory of Belgium, Belgium;
[23]Institute of Space Sciences (ICE, CSIC) and Institut d'Estudis Espacials de Catalunya (IEEC), Spain;
[24]Planetary Science Institute; University of Arizona, AZ-USA;
[25]NASA Johnson Space Center, Houston, TX-USA;

[26]The University of Tokyo, Department of Systems Innovation, School of Engineering, Tokyo, Japan;
[27]Agenzia Spaziale Italiana, Roma, Italy;
[28]Space Science Data Center – ASI, Roma Italy;
[29]Università degli Studi di Napoli "Parthenope", Dipartimento di Scienze & Tecnologie, Centro Direzionale, Napoli, Italy;
[30]Alma Mater Studiorum - Università di Bologna, Dipartimento di Ingegneria Industriale, Forlì, Italy; Alma Mater Studiorum - Università di Bologna, Centro Interdipartimentale di Ricerca Industriale Aerospaziale, Forlì, Italy.
[31]INAF-Istituto di Astrofisica e Planetologia Spaziali, Roma, Italy.

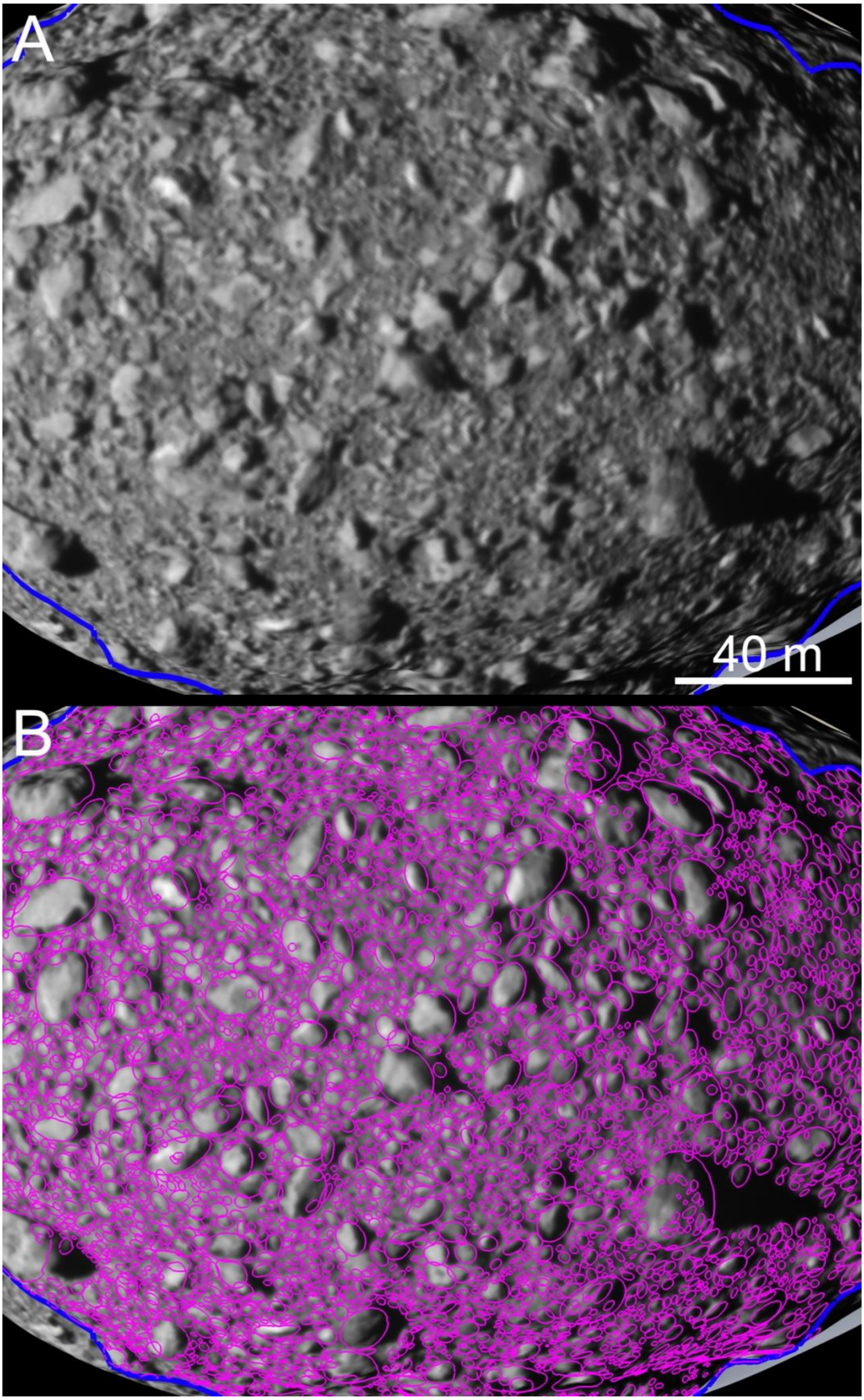

Supplementary Figure 1. Closeup image showing both the uninterpreted boulders (A) and the identified ones, outlined in pink (B). The blue polygon is the study area.

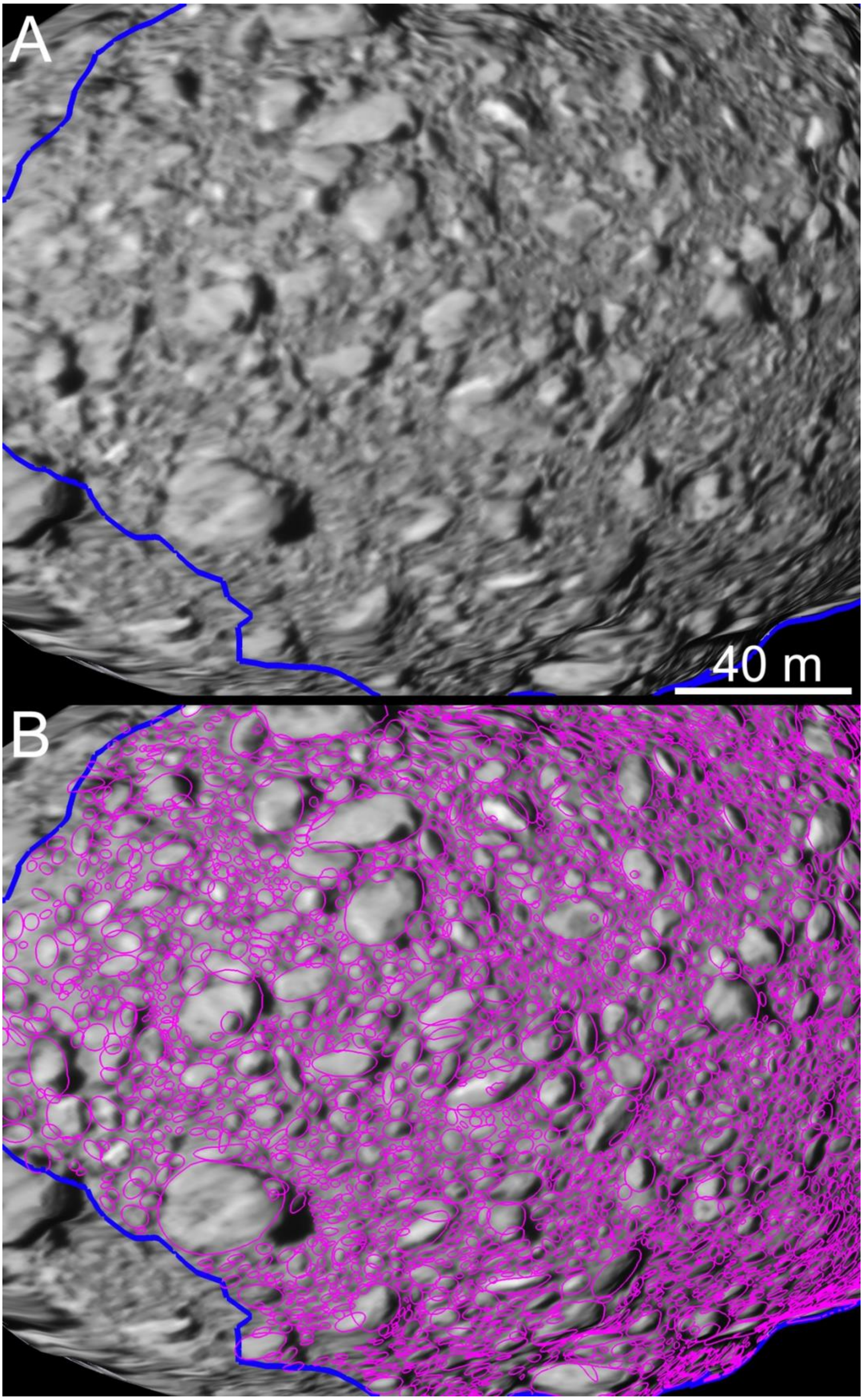

Supplementary Figure 2. Closeup image showing both the uninterpreted boulders (A) and the identified ones, outlined in pink (B). The blue polygon is the study area.

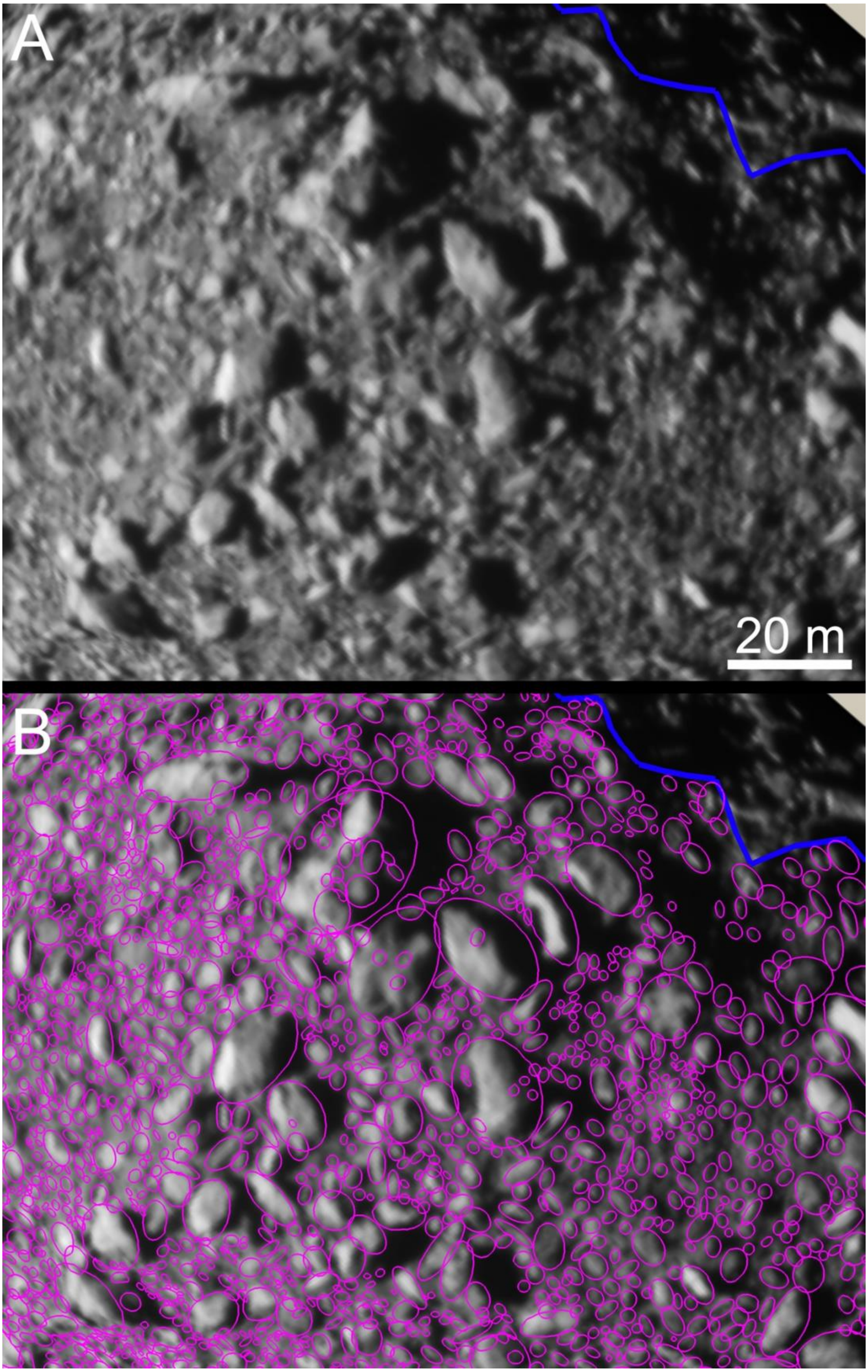

Supplementary Figure 3. Closeup image showing both the uninterpreted boulders (A) and the identified ones, outlined in pink (B). The blue polygon is the study area.
